# Integrative bioinformatics analysis of KPNA2 in six major human cancers

**DOI:** 10.1515/med-2021-0257

**Published:** 2021-03-30

**Authors:** Chaobo Xu, Ming Liu

**Affiliations:** Department of Gastrointestinal Surgery, Lishui People’s Hospital, No. 15 Dazhong Road, Liandu District, Lishui 323000, Zhejiang, China

**Keywords:** KPNA2, bioinformatics, cancer, prognosis

## Abstract

**Background:**

Malignant tumors were considered as the leading causes of cancer-related mortality globally. More and more studies found that dysregulated genes played an important role in carcinogenesis. The aim of this study was to explore the significance of KPNA2 in human six major cancers including non-small cell lung cancer (NSCLC), gastric cancer, colorectal cancer, breast cancer, hepatocellular carcinoma, and bladder cancer based on bioinformatics analysis.

**Methods:**

The data were collected and comprehensively analyzed based on multiple databases. KPNA2 mRNA expression in six major cancers was investigated in Oncomine, the human protein atlas, and GEPIA databases. The mutation status of KPNA2 in the six major cancers was evaluated by online data analysis tool Catalog of Somatic Mutations in Cancer (COSMIC) and cBioPortal. Co-expressed genes with KPNA2 were identified by using LinkedOmics and made pairwise correlation by Cancer Regulome tools. Protein-protein interaction (PPI) network relevant to KPNA2 was constructed by STRING database and KEGG pathway of the included proteins of the PPI network was explored and demonstrated by circus plot. Survival analysis-relevant KPNA2 of the six cancers was performed by GEPIA online data analysis tool based on TCGA database.

**Results:**

Compared with paired normal tissue, KPNA2 mRNA was upregulated in all of the six types of cancers. KPNA2 mutations, especially missense substitution, were widely identified in six cancers and interact with different genes in different cancer types. Genes involved in PPI network were mainly enriched in p53 signaling pathway, cell cycle, viral carcinogenesis, and Foxo signaling pathway. KPNA2 protein was mainly expressed in nucleoplasm and cytosol in cancer cells. Immunohistochemistry assay indicated that KPNA2 protein was also positively expressed in nucleoplasm with brownish yellow staining. Overall survival (OS) and progression free survival (PFS) were different between KPNA2 high and low expression groups.

**Conclusions:**

KPNA2 was widely dysregulated and mutated in carcinomas and correlated with the patients prognosis which may be potential target for cancer treatment and biomarker for prognosis.

## 1 Introduction

Cancer is the leading cause of death globally. According to the cancer statistical analysis in year 2018, 9.6 million deaths and 18.1 million new cases of all types of cancers were estimated [1]. Cancer had posed a heavy burden not only for the human beings, but also for the governments and health provides. Lung cancer especially non-small cell lung cancer (NSCLC), gastric cancer, colorectal cancer, breast cancer, liver hepatic cancer, and bladder cancer were the most common carcinomas diagnosed clinically with high incidence and mortality [2,3]. Although the above six cancers had high incidence and poor prognosis, the general carcinogenesis was not completely cleart. In recent years, with the development of biology and life science, more and more evidence had made it clear that the driving genes had played an important role in the carcinogenesis and pathways [4]. The driving genes including oncogenes and tumor suppressor genes involved in the cell division, apoptosis [5], proliferation [6], and migration may act as the general phenotype of malignant carcinoma.

Karyopherin subunit alpha 2 (KPNA2) is one of the important members of karyophenin family [7]. It has three functional domains: N-terminal Importin β binding domain, central domain, and a short acid C-terminal. The central domain contains the nuclear localization signal (NLS) binding site and CAS binding site [8,9]. In the cytoplasm, Karyopherin subunit alpha 2 can recognize and bind nucleophilic NLS, while Importin β will combine with karyophenin to form NLS-α/β complex, and then enter the nucleus through the nuclear pore complex under the energy provided by RanGTP enzyme [8]. KPNA2 is not expressed or low expressed in normal tissues, but it is upregulated in some types of carcinoma such as breast cancer [10,11] and ovarian cancer [12]. However, seldom studies focused on the KPNA2 expression, mutation, and as prognostic marker for pan-cancer. In the present work, we investigated KPNA2 mRNA expression, mutation, and prognostic significance in six major carcinomas through well-known online databases.

## 2 Methods

### 2.1 KPNA2 mRNA expression analysis

KPNA2 mRNA expression level of human normal tissues and multiple cancers was identified in the human protein atlas database (https://www.proteinatlas.org/) with data original from HPA, GTEx, and FANTOM5 project. KPNA2 mRNA expression level between cancer tissue and paired normal tissue was further validated by Oncomine database (https://www.oncomine.org/resource/login.html) [13] and GEPIA online data analysis tool (http://gepia.cancer-pku.cn/detail.php) with data origin from the TCGA database.

### 2.2 KPNA2 gene mutation analysis

KPNA2 gene mutation was analyzed through the cBio cancer genomics portal (http://cbioportal.org) with the data origin from the TCGA database. The mutation frequency of nonsense substitution, missense substitution, synonymous substitution, inframe insertion, frameshift, etc. was identified and expressed by pie plot. The single nucleotide mutation of KPNA2 was also screened by catalogue of somatic mutation in cancer (COSMIC) (https://cancer.sanger.ac.uk/cosmic/) online data analysis tool [14].

### 2.3 Genome-wide association of KPNA2 mRNA in cancer analysis

The expression of KPNA2 gene and its correlation with other genes of the six cancer types was expressed by the circus plots generated from the Cancer Regulome tools and data (http://explorer.cancerregulome.org/). Co-expressed genes were clustered and demonstrated by the heat map generated from LinkedOmics database (http://www.linkedomics.org/login.php) [15]. The top positive and negative correlated genes with KPNA2 were identified and made Pearson correlations test.

### 2.4 PPI network construction and KEGG pathway enrichment

The protein–protein interaction (PPI) network relevant to KPNA2 was constructed by the STRINIG database (http://string-db.org/cgi/input.pl) [16]. The PPI network was constructed under the condition of: network type: full STRING network type; meaning of network edges: evidence; minimum required interaction score: 0.40. The genes included in the PPI network were identified and made KEGG pathway enrichment demonstrated by circus plot.

### 2.5 Survival analysis

According to the median expression level of KPNA2 mRNA, cancer patients were divided into high expression (≥median expression) group and low expression group. The progression free survival (PFS) and overall survival (OS) were compared between KPNA2 high and low expression groups of the six cancer types and demonstrated by survival curve [17].

### 2.6 KPNA2 protein expression analysis

KPNA2 protein expression in tumor cell lines and cancer tissues was detected by immunofluorescent staining and immunohistochemistry assay in the human protein atlas database (https://www.proteinatlas.org/).

### 2.7 Statistical analysis

The data were analyzed based on the relevant databases or online data analysis tool.

## 3 Results

### 3.1 KPNA2 mRNA expression in normal and tumor tissue

**Figure 1:**
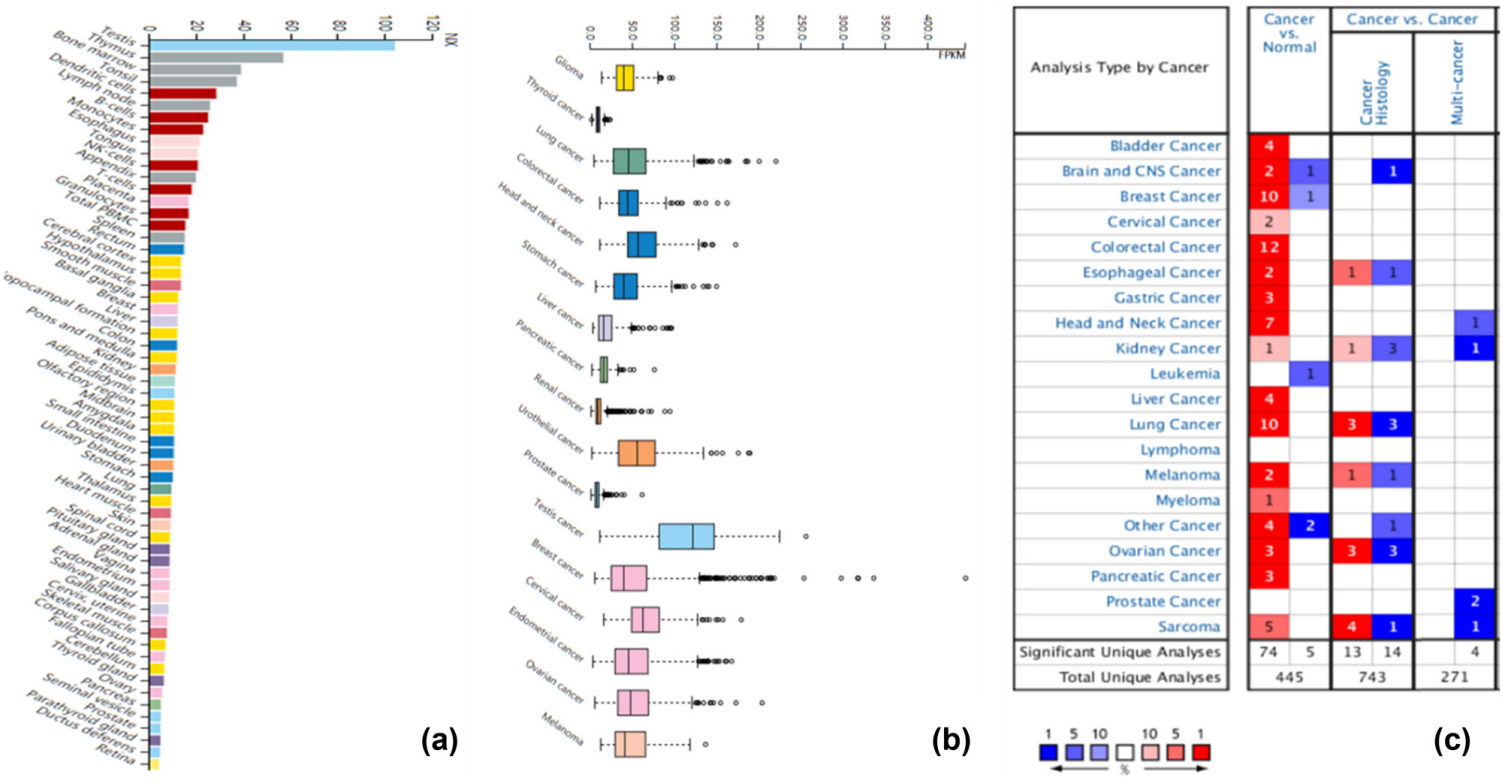
KPNA2 expression analysis. (a) KPNA2 expression in across human body tissues, (b) KPNS2 expression across carcinomas, (c) KPNA2 expression between cancer tissue and paired normal tissues based on oncoming database.

**Figure 2:**
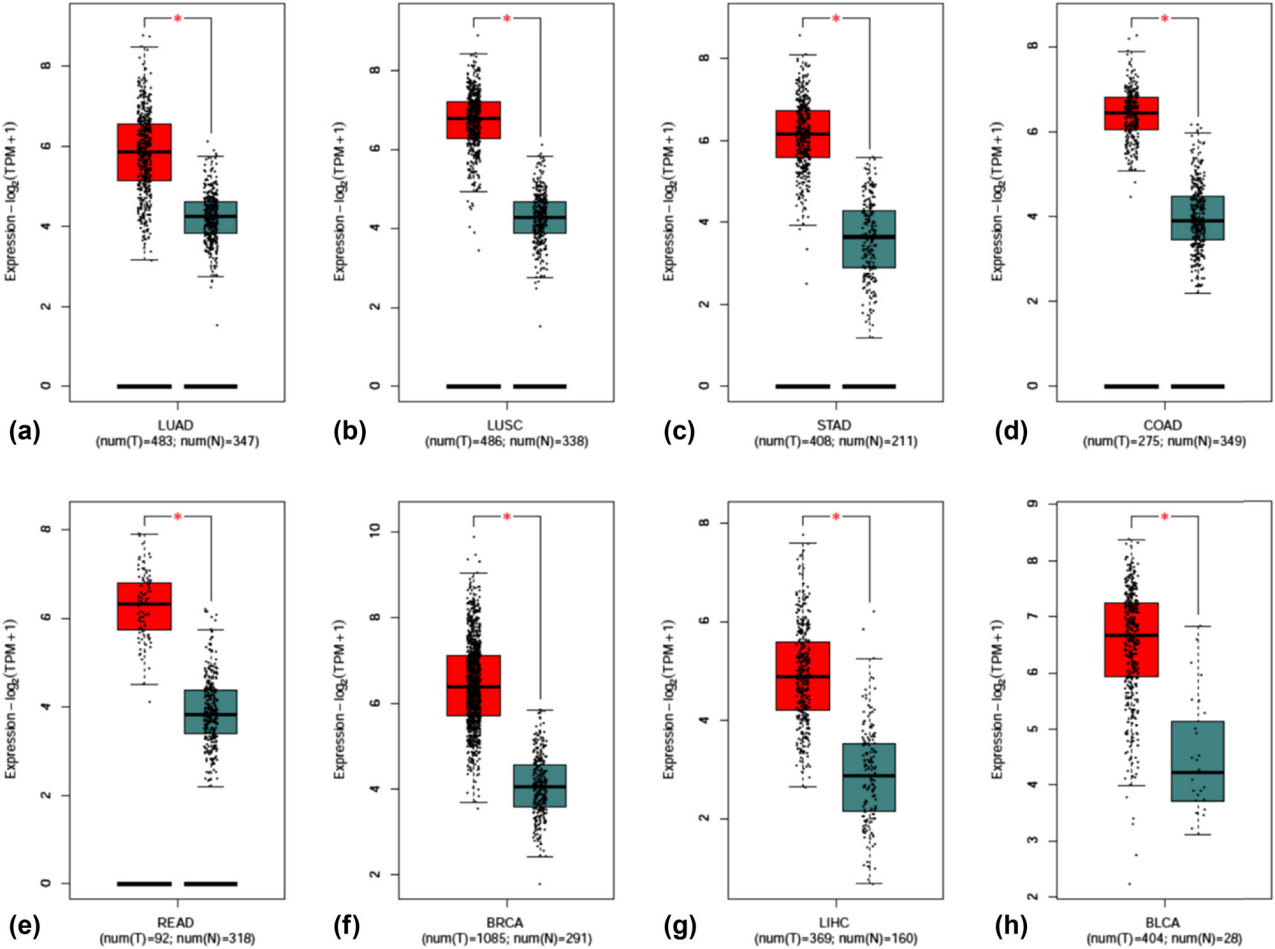
Scatter plot of KPNA2 mRNA expression between paired normal tissue and cancer tissue. (a) Lung adenocarcinoma, (b) lung squamous cell carcinoma, (c) gastric cancer, (d) colon cancer, (e) Rectal cancer, (f) breast cancer, (g) hepatocellular carcinoma, (h) bladder cancer.

KPNA2 mRNA expression in all human body tissues is demonstrated in Figure 1a. The expression level was quite different across different tissues. KPNA2 mRNA expression levels in different type cancers are showed in Figure 1b, which indicated that the expression level across different cancers was not obviously different. KPNA2 was upregulated in cancer tissue compared with paired normal tissue in all the six major cancers based on Oncomine database (Figure 1c) and GEPIA with statistical difference (*p* < 0.05) (Figure 2).

### 3.2 KPNA2 mutation analysis

**Figure 3:**
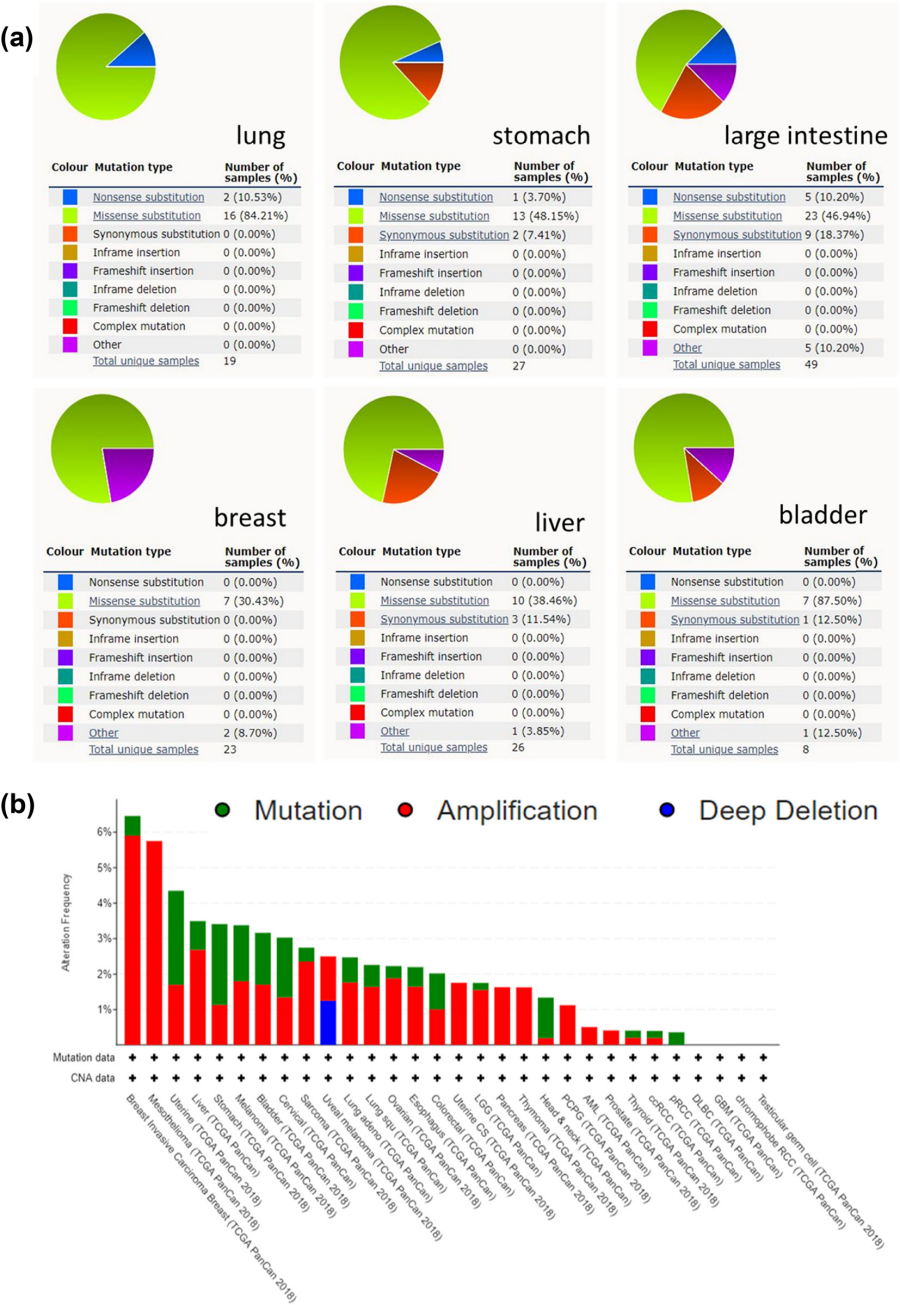
KPNA2 mutation analysis. (a) Pie plot of the KPNA2 mutation frequency of the six major cancers. (b) Bar chart of KPNA2 mutation in pan-cancers based on TCGA database.

**Figure 4:**
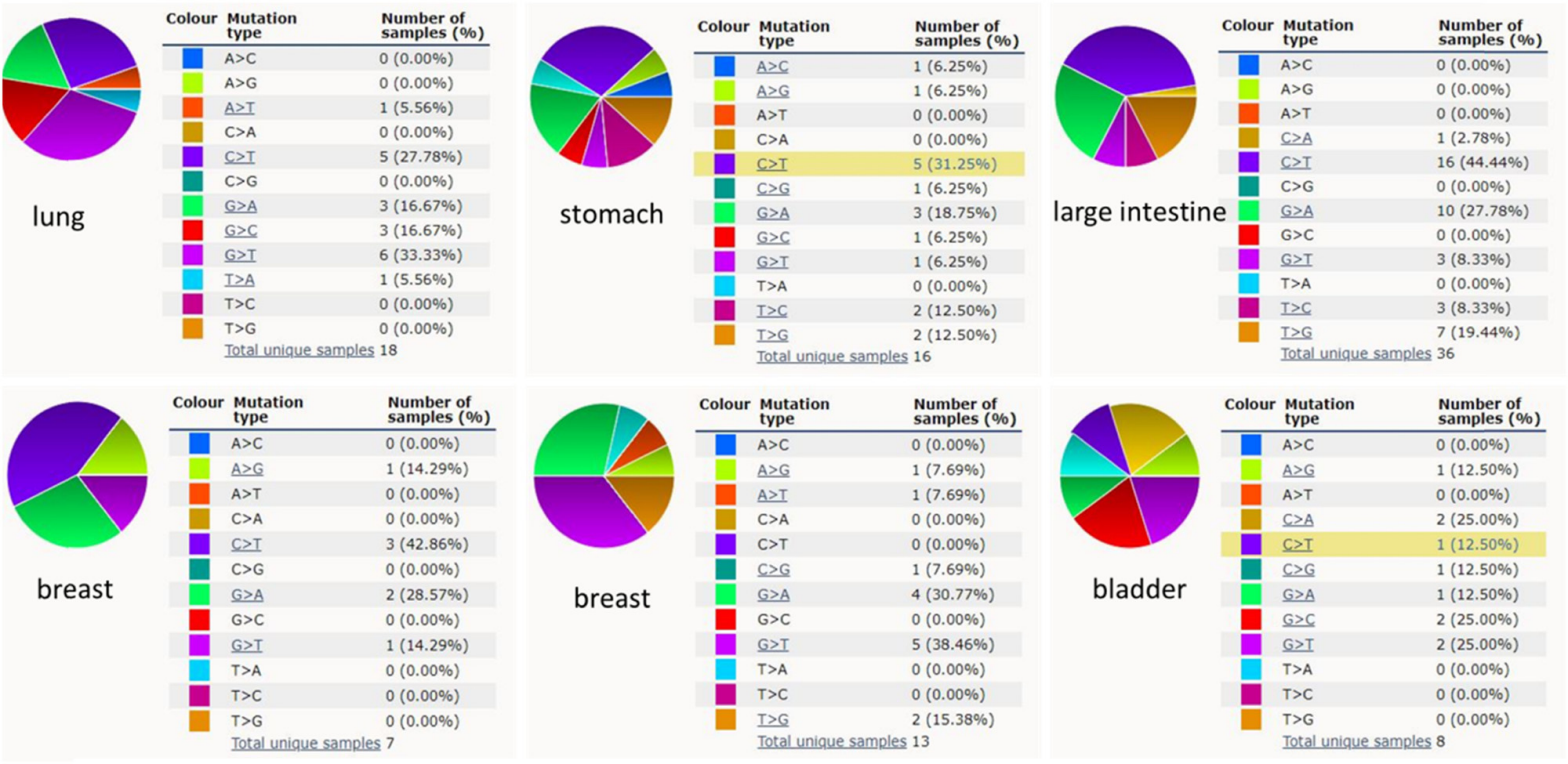
KPNA2 single nucleotide mutation analysis.

KPNA2 mutation status of the six cancers was evaluated by online data analysis tool Catalog of Somatic Mutations in Cancer (COSMIC) and cBioPortal. KPNA2 mutations were widely identified in six major cancers and interact with different genes in different cancer types. Missense substitution was found in lung cancer (84.21%), gastric cancer (48.15%), colorectal cancer (46.94%), breast cancer (30.43%), liver hepatic cancer (38.46%), and bladder cancer (87.50%). Other major mutations including nonsense substitution and synonymous substitution were also identified in the six major cancers (Figure 3a). For pan-cancers analysis, KPNA2 highly mutated in uterine carcinoma, stomach cancer, cervical cancer, breast cancer, etc. based on TCGA database (Figure 3b). For single nucleotide mutation, C > T and G > T were most common in the KPNA2 coding strand, both of which were identified in the six major cancer types. And other kinds of single nucleotide mutations were rare in TCGA cancer samples of the six cancer types (Figure 4).

### 3.3 Genome-wide association of KPNA2 in cancer

**Figure 5:**
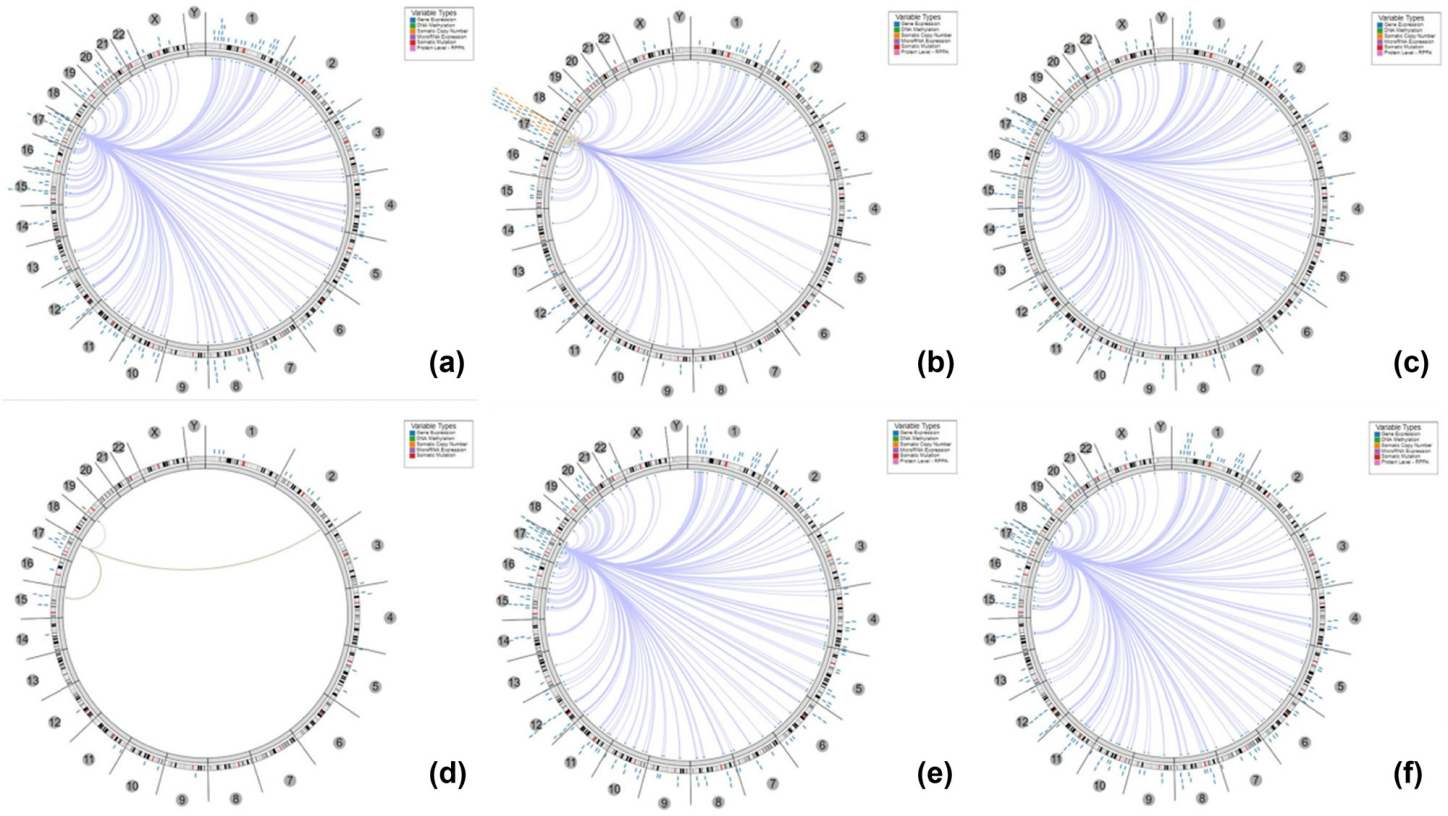
The circus plot demonstrated the associations between KPNA2 and other genes. The edges in the center connecting the features (with genomic coordinates) displayed around the perimeter. The outer ring displays cytogenetic bands. The inner ring displays associations that contain features lacking genomic coordinates. (a) Non-small cell lung cancer, (b) gastric cancer, (c) colorectal cancer, (d) breast cancer, (e) liver hepatic cancer, (f) bladder cancer.

Based on the association among genes, DNA methylation, somatic copy number, somatic mutation, and protein level, circus plots were drawn to display the interrelation between KPNA2 and other genes. According to the data from TCGA, KPNA2 was associated with other genes that could be detected in NSCLC, gastric cancer, colorectal cancer, liver hepatic cancer, and bladder cancer (Figure 5).

### 3.4 Co-expressed genes with KPAN2 in six major cancers

**Figure 6:**
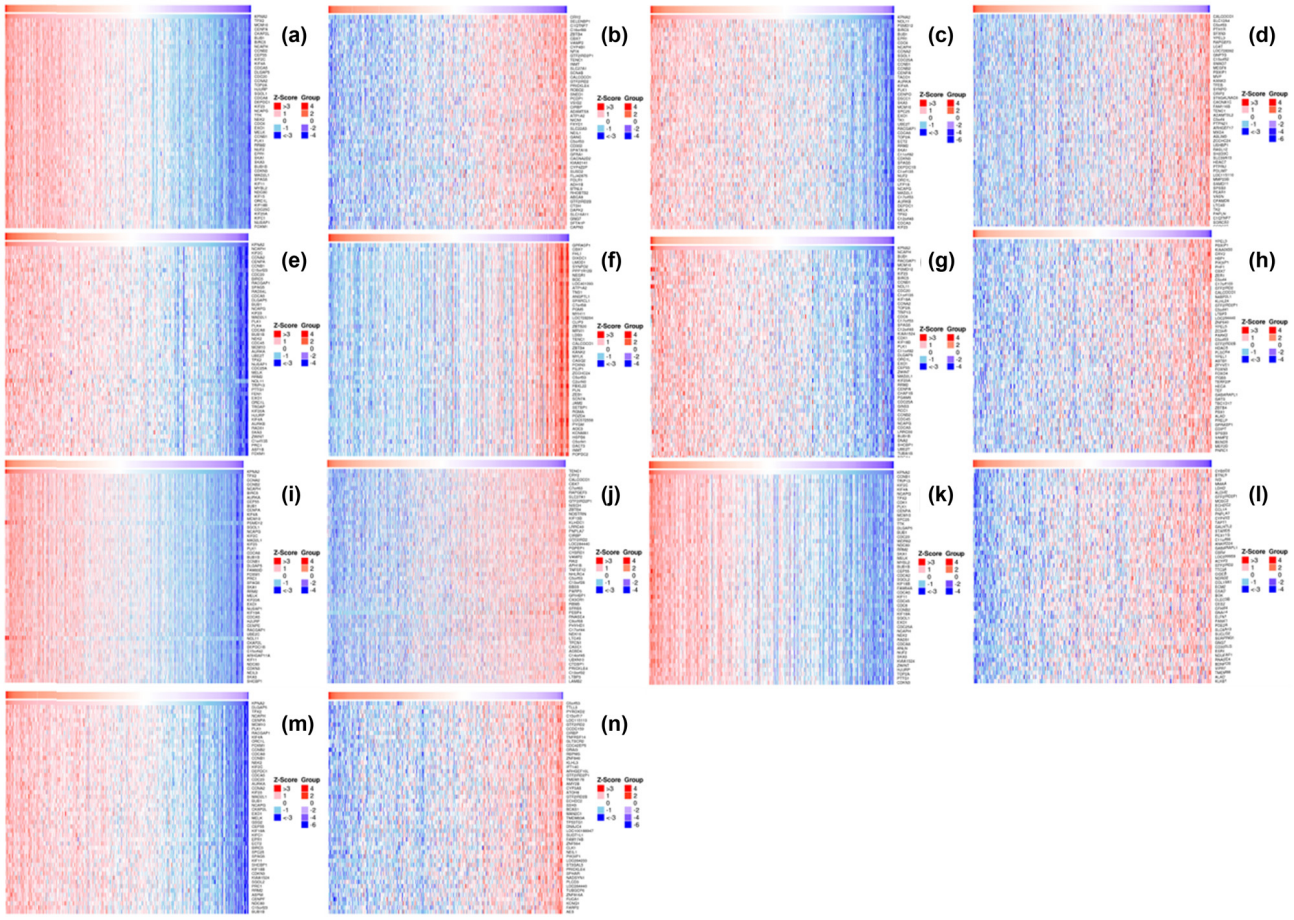
Heat map of co-expressed genes with KPAN2 in six major cancers. (a) Positive co-expressed genes with KPAN2 in lung adenocarcinoma, (b) negative co-expressed genes with KPAN2 in lung adenocarcinoma, (c) positive co-expressed genes with KPAN2 in lung squamous carcinoma, (d) negative co-expressed genes with KPAN2 in lung squamous carcinoma, (e) positive co-expressed genes with KPAN2 in gastric cancer, (f) negative co-expressed genes with KPAN2 in gastric cancer, (g) positive co-expressed genes with KPAN2 in colorectal cancer, (h) negative-expressed genes with KPAN2 in colorectal cancer, (i) positive co-expressed genes with KPAN2 in breast cancer, (j) negative co-expressed genes with KPAN2 in breast cancer, (k) positive co-expressed genes with KPAN2 in liver hepatic cancer, (l) negative co-expressed genes with KPAN2 in breast cancer, (m) positive co-expressed genes with KPAN2 in bladder cancer, (n) negative co-expressed genes with KPAN2 in bladder cancer.

**Figure 7:**
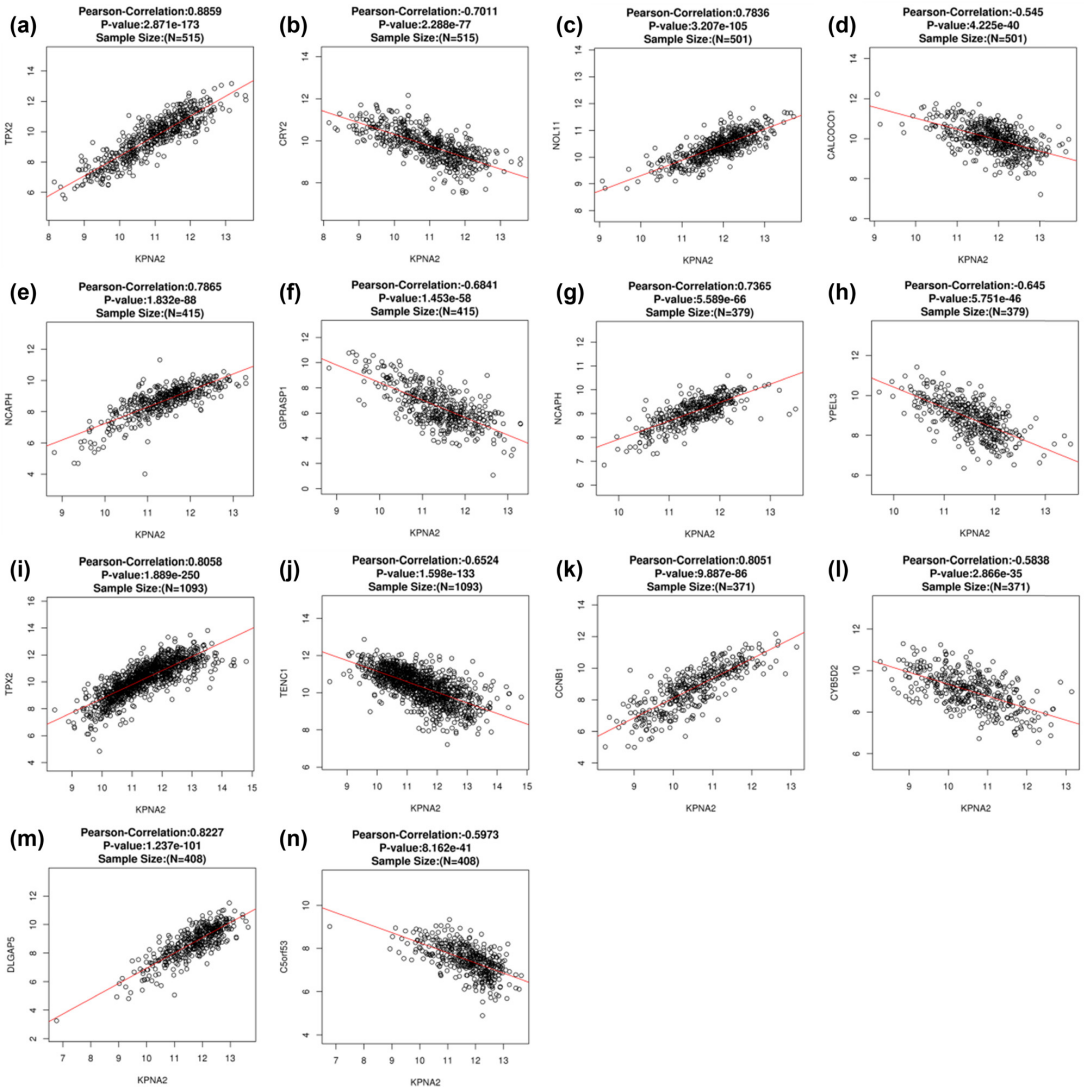
The top positive and negative correlated genes with KPAN2 in six major cancers. (a) Positive co-expressed genes with KPAN2 in lung adenocarcinoma, (b) negative co-expressed genes with KPAN2 in lung adenocarcinoma, (c) positive co-expressed genes with KPAN2 in lung squamous carcinoma, (d) negative co-expressed genes with KPAN2 in lung squamous carcinoma, (e) positive co-expressed genes with KPAN2 in gastric cancer, (f) negative co-expressed genes with KPAN2 in gastric cancer, (g) positive co-expressed genes with KPAN2 in colorectal cancer, (h) negative-expressed genes with KPAN2 in colorectal cancer, (i) positive co-expressed genes with KPAN2 in breast cancer, (j) negative co-expressed genes with KPAN2 in breast cancer, (k) positive co-expressed genes with KPAN2 in liver hepatic cancer, (l) negative co-expressed genes with KPAN2 in breast cancer, (m) positive co-expressed genes with KPAN2 in bladder cancer, (n) negative co-expressed genes with KPAN2 in bladder cancer.

The co-expressed genes with KPAN2 in six cancers were demonstrated with the heatmap (Figure 6). The top positive and negative correlated genes with KPAN2 in six cancers are showed in Figure 7.

### 3.5 PPI network of KPNA2

**Figure 8:**
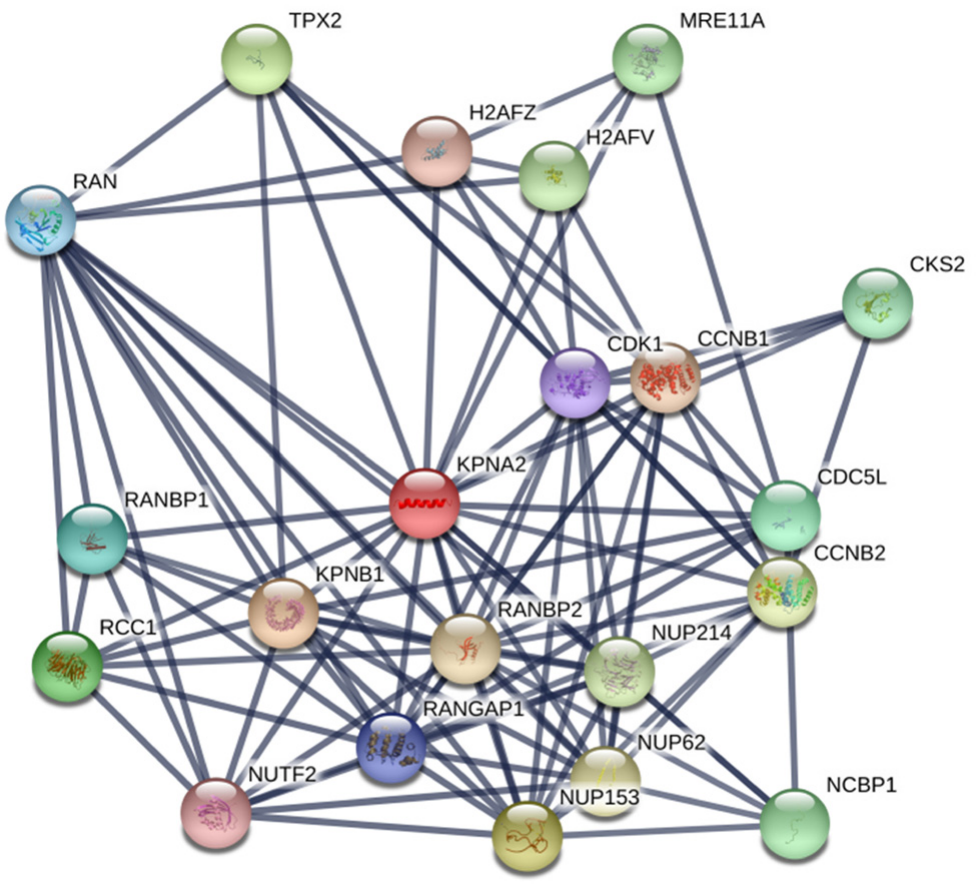
The PPI network included KPNA2 and relevant genes

Twenty genes were included in the PPI network with the edges of 105 and local clustering coefficient of 0.713, which indicated that the PPI enrichment was significant with statistical difference (*p* < 0.001) (Figure 8).

### 3.6 KEGG pathway relevant KPNA2

**Figure 9:**
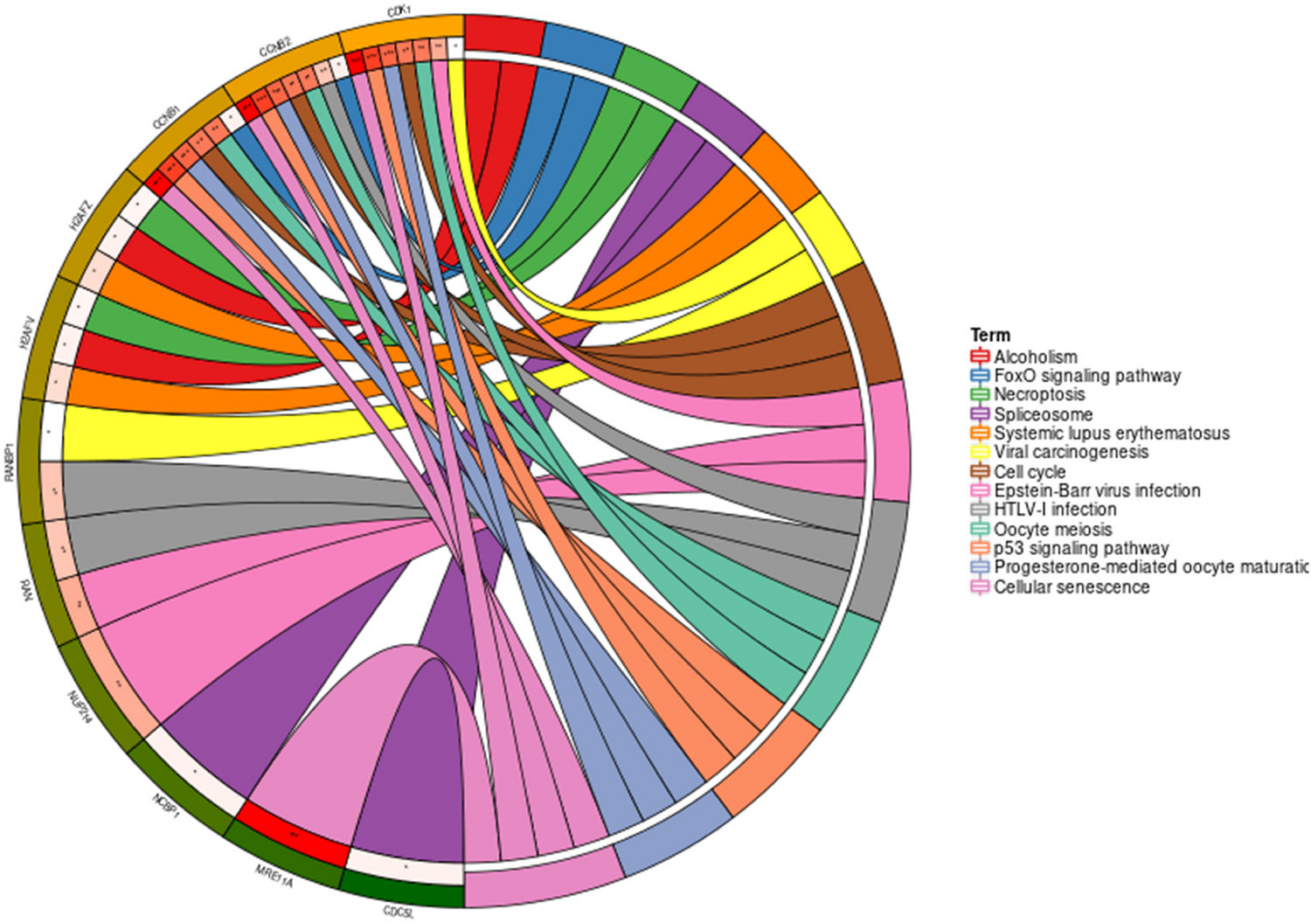
Circus plot of KEGG pathway enrichment of genes that are relevant to KPNA2

Genes involved in PPI network were mainly enriched in p53 signaling pathway, cell cycle, viral carcinogenesis, Foxo signaling pathway, etc. (Figure 9).

### 3.7 KPNA2 mRNA level and patients’ prognosis

**Figure 10:**
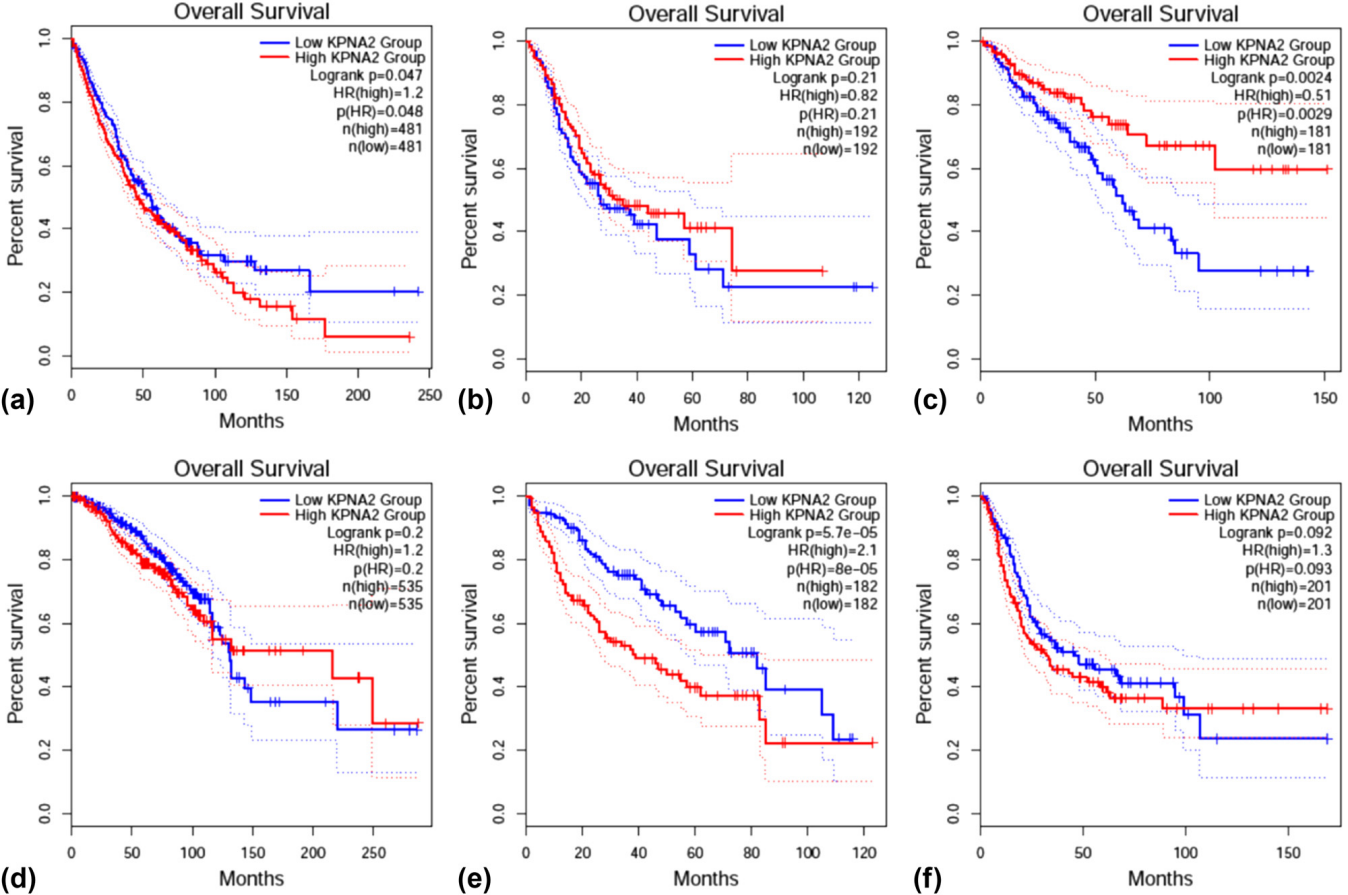
Overall survival (OS) between KPNA2 between high and low expression groups in six major cancers. (a) NSCLC, (b) gastric cancer, (c) colorectal cancer, (d) breast cancer, (e) liver hepatic cancer, (f) bladder cancer.

**Figure 11:**
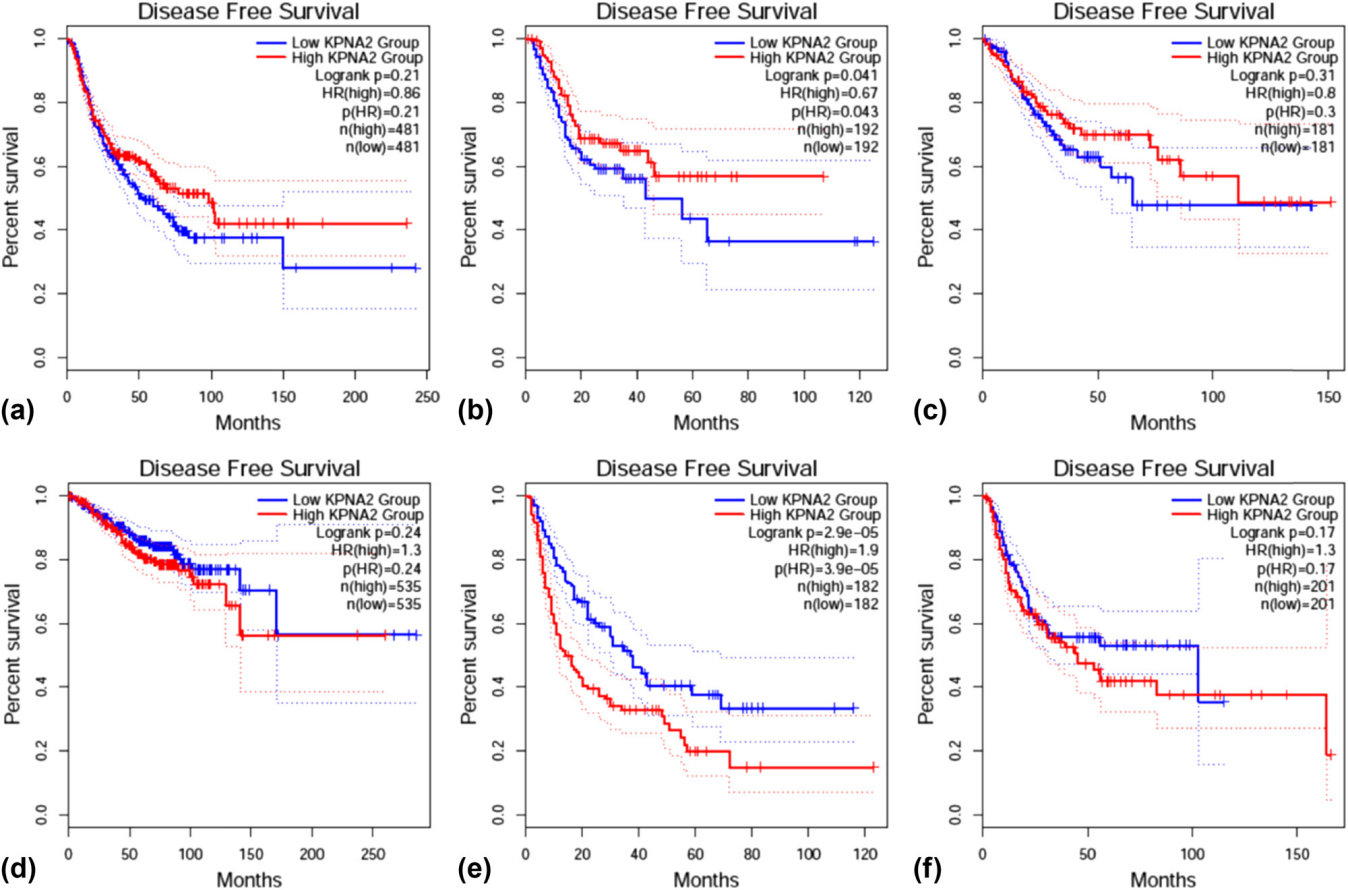
Disease free survival (DFS) between KPNA2 between high and low expression groups in six major cancers. (a) NSCLC, (b) gastric cancer, (c) colorectal cancer, (d) breast cancer, (e) liver hepatic cancer, (f) bladder cancer.

OS was statistically different between KPNA2 mRNA high and low expression groups in NSCLC (HR = 1.2, *p* < 0.05), colorectal cancer (HR = 0.51, *p* < 0.01), and liver hepatic carcinoma (HR = 2.1, *p* < 0.001) (Figure 10). For disease free survival (DFS), the statistical difference was found in gastric cancer (HR = 0.67, *p* < 0.05) and liver hepatic cancer (HR = 1.9, *p* < 0.001) (Figure 11).

### 3.8 KPNA2 protein expression

**Figure 12:**
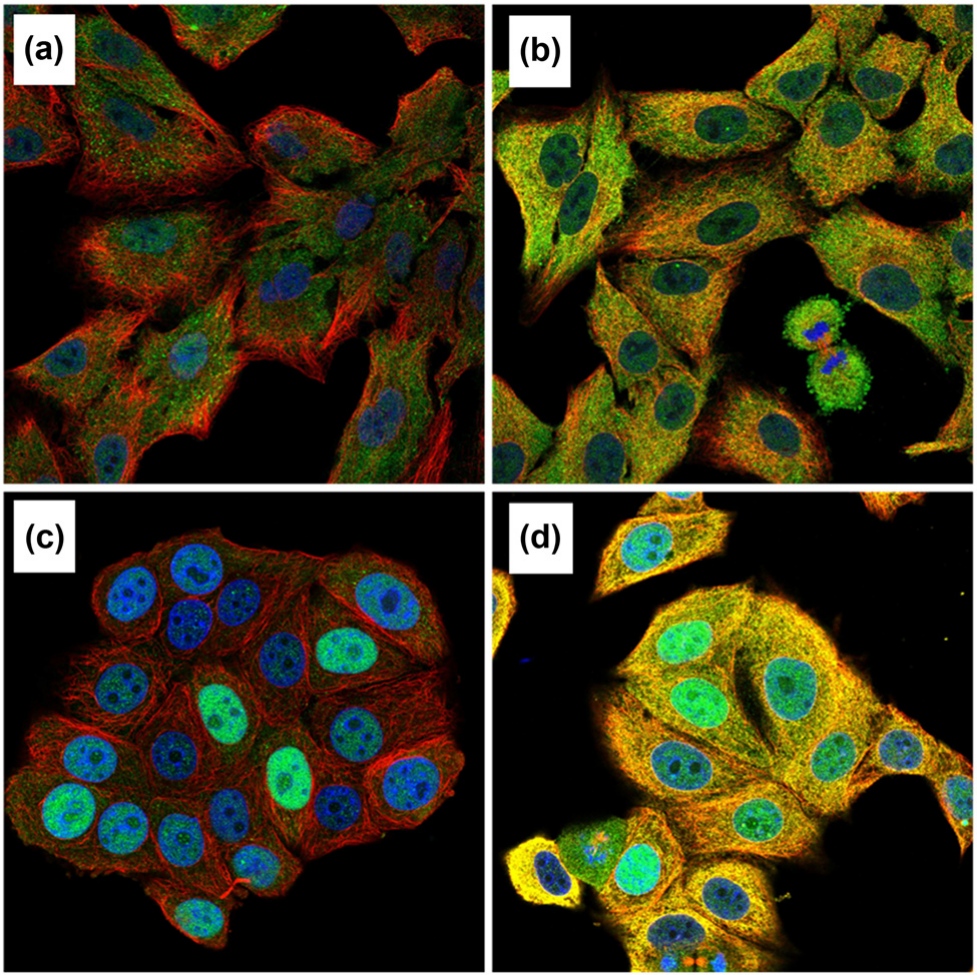
KPNA2 protein was mainly localized to the nucleoplasm and cytosol in cancer cells with blue staining. (a) Immunofluorescent staining of human lung cancer cell line A549 shows localization to nucleoplasm in antibody + nucleus + microtubule channels. (b) Immunofluorescent staining of human lung cancer cell line A549 shows localization to nucleoplasm in antibody + nucleus + microtubule + ER channels. (c) Immunofluorescent staining of human breast cancer cell line MCF7 shows localization to nucleoplasm in antibody + nucleus + microtubule channels. (d) Immunofluorescent staining of human breast cancer cell line MCF7 shows localization to nucleoplasm in antibody + nucleus + microtubule + ER channels.

**Figure 13:**
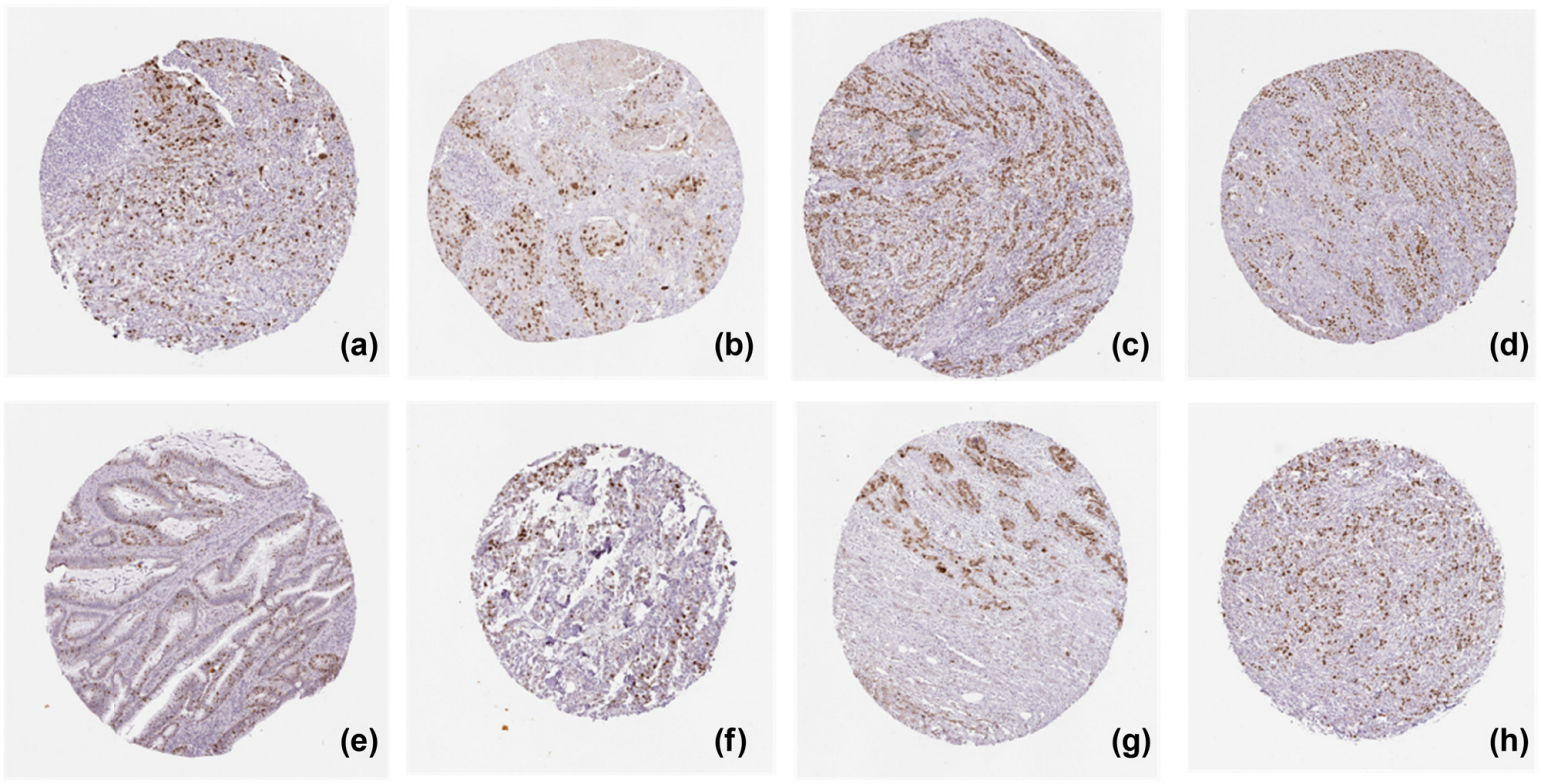
KPNA2 protein positive expression in six major cancers detected by Immunohistochemistry assay. (a) Lung adenocarcinoma, (b) lung squamous cell carcinoma, (c) gastric cancer, (d) colon cancer, (e) rectal cancer, (f) breast cancer, (g) liver hepatic carcinoma, (h) bladder cancer.

KPNA2 protein was mainly expressed in nucleoplasm and cytosol in cancer cells detected by immunofluorescence assay (Figure 12). Immunohistochemistry assay indicated that KPNA2 protein was also positively expressed in nucleoplasm with Brownish yellow staining (Figure 13).

## 4 Discussion

**Figure 14:**
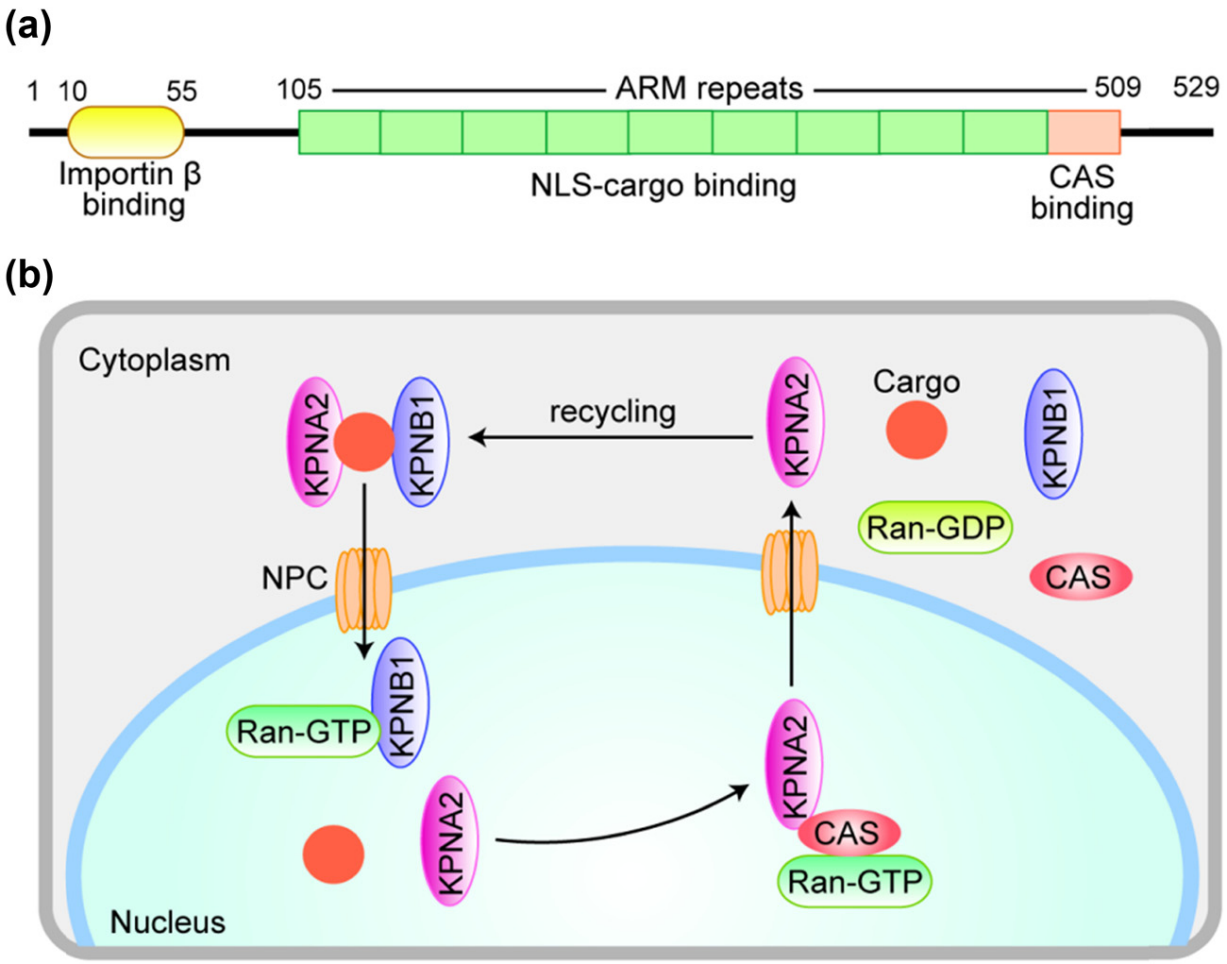
A diagrammatic representation of KPNA2 protein structure (a) and the molecular mechanism of KPNA2 nucleoplasmic recirculation (b). (a) The N-terminus is the Importin β (KPNB1) binding domain, ensuring that KPNA2 can only be translocated into the nucleus while simultaneously combining KPNB1 and cargo protein. The central region consists of 10 armadillo (ARM) repeats, including two NLS-cargo binding sites. The last ARM repeat mediates CAS binding. (b) KPNB1 brings a complex of KPNA2 and Cargo protein into the nucleus via NPC and binds to RanGTP to release KPNA2 and cargo proteins into the nucleus. Then KPNB1 returns directly to the cytoplasm. KNPA2 returns to the cytoplasm with the help of another transporter, CAS, for the next cycle.

The structural nuclear transporter family of KPNA2 includes the input protein family and the output protein family [18]. It mainly mediates proteins with molecular weight greater than 40 kDa through nuclear pore complexes (NPC). The input protein family includes karyophenin α family and import β family. There are seven members in karyophenin α family, of which karyophenin α 2 (KPNA2) is one of the most important members. KPNA2 gene is located in chromosome 17q23-q24 in human being and its encoded protein contains 529 amino acids, with a molecular weight of 58 kDa [9,19]. The N-terminal is the Importin β binding domain, which has self-inhibition function, so that KPNA2 can only bind to importin at the same time β and cargo molecules can only be translocated to the nucleus [20,21]. The central region is composed of ten arm repeat sequences, including 2 NLS binding sites, which can bind to the nucleoprotein with NLS, and the 10th arm sequence can bind to CAS, which is responsible for kpna2 nucleoplasm recycling; the function of C-terminal is not completely clear yet (Figure 14a).

The classical nuclear protein input is regulated by heterodimer composed of importin β and karyophenin α. Karyophenin α protein can recognize and bind NLS of cargo protein. Importin β brings the complex composed of karyophenin α and nucleoprotein into the nucleus through NPC and combines with RanGTP to form protein complex in the nucleus, so as to release karyophenin α and nucleoprotein into the nucleus, and then importin β returns directly to the cytoplasm, while karyophenin α returns to the cytoplasm with the help of CAS [22] (Figure 14b).

KPNA2 is a member of the karyopherin family. Given its function in nucleocytoplasmic transport, KPNA2 mediates the translocation of various proteins and is involved in numerous cellular processes, such as cellular differentiation, proliferation, apoptosis, transcriptional regulation, immune response, and viral infection. Recently, several studies have demonstrated that KPNA2 was upregulated in multiple malignancies [23,24,25]. Its aberrant expression was often associated with adverse outcomes in affected patients, indicating that KPNA2 played a significant role in carcinogenesis and tumor progression [23,26,27]. These findings were supported by previous studies, which reported that KPNA2 may have a functional role in the malignant transformation of cells.

As an intranuclear transporter, KPNA2 is involved in cell differentiation, proliferation and apoptosis, transcriptional regulation, immune response, and virus infection. More importantly, many studies have found that Pna2 was involved in tumor progression by regulating the nuclear translocation of tumor-related proteins. It has been reported that: (1) KPNA2 was involved in the nuclear translocation of many tumor-related transcription factors, including E2F1 and pleomorphic adenoma, two members of the zinc finger plag family Gene1, plag1, [28] and lot1 [29] and BTB/POZ transcription factor kaiso [30], etc.; (2) KPNA2 mediates Rac-1 nuclear translocation [31]; Rac-1 participates in tumor formation by participating in cell cycle, cell adhesion, and migration; (3) KPNA2 mediates the entry of cell cycle regulatory protein CHK2 into the nucleus, and overexpression of KPNA2 leads to increased nuclear input of CHK2 [32]; (4) KPNA2 participates in breast cancer suppression – BRCA1 has the function of DNA repair and cell cycle monitoring, which affects the process of tumor formation; (5) KPNA2 participates in the entry of NBS1, which is a kind of DNA repair complex protein and also involved in tumor formation [33].

In our present work, we noticed that KPNA2 mRNA was upregulated in all of the six types of cancers compared with paired normal tissue. KPNA2 mutations, especially missense substitution, were widely identified in six cancers and interact with different genes in different cancer types. Genes involved in PPI network were mainly enriched in p53 signaling pathway, cell cycle, viral carcinogenesis, and Foxo signaling pathway. Immunohistochemistry assay indicated that KPNA2 protein was also positively expressed in nucleoplasm with brownish yellow staining. OS and PFS were generally different between KPNA2 high and low expression groups, which may be a potential biomarker for cancer prognosis. However, there are still limitations for the present work. First, there may be potential heterogeneity across the six cancer types and the results need further validation. Second, the results of the present were mainly based on data mining from relevant databases which should be further validated by local data or experiments.

## 5 Conclusion

KPNA2, as an intranuclear transport protein, participates in a variety of biological activities through the transport function of nucleoplasm, and its role in tumor development has attracted more and more attention. In view of the abnormal expression of KPNA2 in most cancer tissues and serum of the cancer patients, and related to the proliferation, migration, and invasion of tumor cells, KPNA2 can be used as a potential biomarker for prognosis. However, the mechanism of KPNA2 in tumorigenesis and progression is not completely clear yet and needs further investigation.


**Ethics approval and consent to participate:** Not applicable.

## References

[1] Bray F, Ferlay J, Soerjomataram I, Siegel RL, Torre LA, Jemal A. Global cancer statistics 2018: GLOBOCAN estimates of incidence and mortality worldwide for 36 cancers in 185 countries. CA Cancer J Clin. 2018;68:394–424. 10.3322/caac.21492 30207593

[2] Siegel RL, Miller KD, Jemal A. Cancer statistics, 2019. CA Cancer J Clin. 2019;69:7–34. 10.3322/caac.21551 30620402

[3] Siegel RL, Miller KD, Jemal A. Cancer statistics, 2020. CA Cancer J Clin. 2020;70:7–30. 10.3322/caac.21590 31912902

[4] Johnson LA, June CH. Driving gene-engineered T cell immunotherapy of cancer. Cell Res. 2017;27:38–58. 10.1038/cr.2016.154 PMC5223234 28025979

[5] Zhang KJ, Qian J, Wang SB, Yang Y. Targeting gene-viro-therapy with AFP driving apoptin gene shows potent antitumor effect in hepatocarcinoma. J Biomed Sci. 2012;19:20. 10.1186/1423-0127-19-20 PMC3311074 22321574

[6] Cui Y, Zhang C, Ma S, Guo W, Cao W, Guan F. CASC5 is a potential tumour driving gene in lung adenocarcinoma. Cell Biochem Funct. 2020;38(6):733–42. 10.1002/cbf.3540 32283571

[7] Kelley JB, Talley AM, Spencer A, Gioeli D, Paschal BM. Karyopherin alpha7 (KPNA7), a divergent member of the importin alpha family of nuclear import receptors. BMC Cell Biol. 2010;11:63. 10.1186/1471-2121-11-63 PMC2929220 20701745

[8] Yasuhara N, Kumar PK. Aptamers that bind specifically to human KPNA2 (importin-α1) and efficiently interfere with nuclear transport. J Biochem. 2016;160:259–68. 10.1093/jb/mvw032 PMC7110145 27154959

[9] Dörr SN, Schlicker MN, Hansmann IN. Genomic structure of karyopherin alpha2 ( KPNA2) within a low-copy repeat on chromosome 17q23-q24 and mutation analysis in patients with Russell-Silver syndrome. Hum Genet. 2001;109:479–86. 10.1007/s004390100605 11735022

[10] Alshareeda AT, Negm OH, Green AR, Nolan CC, Tighe P, Albarakati N, et al. A KPNA2 is a nuclear export protein that contributes to aberrant localisation of key proteins and poor prognosis of breast cancer. Br J Cancer. 2015;112:1929–37. 10.1038/bjc.2015.165 PMC4580386 25989275

[11] Dankof A, Fritzsche FR, Dahl E, Pahl S, Wild P, Dietel M, et al. KPNA2 protein expression in invasive breast carcinoma and matched peritumoral ductal carcinoma in situ. Virchows Arch. 2007;451:877–81. 10.1007/s00428-007-0513-5 17899179

[12] Huang L, Zhou Y, Cao XP, Lin JX, Zhang L, Huang ST, et al. KPNA2 is a potential diagnostic serum biomarker for epithelial ovarian cancer and correlates with poor prognosis. Tumour Biol. 2017;39:1010428317706289. 10.1177/1010428317706289 28651492

[13] Rhodes DR, Yu J, Shanker K, Deshpande N, Varambally R, Ghosh D, et al. ONCOMINE: a cancer microarray database and integrated data-mining platform. Neoplasia. 2004;6:1–6. 10.1016/s1476-5586(04)80047-2 PMC1635162 15068665

[14] Li QT, Huang ZZ, Chen YB, Yao HY, Ke ZH, He XX, et al. Integrative analysis of Siglec-15 mRNA in human cancers based on data mining. J Cancer. 2020;11:2453–64. 10.7150/jca.38747 PMC7066007 32201516

[15] Vasaikar SV, Straub P, Wang J, Zhang B. LinkedOmics: analyzing multi-omics data within and across 32 cancer types. Nucleic Acids Res. 2018;46:D956–63. 10.1093/nar/gkx1090 PMC5753188 29136207

[16] Szklarczyk D, Gable AL, Lyon D, Junge A, Wyder S, Huerta-Cepas J, et al. STRING v11: protein–protein association networks with increased coverage, supporting functional discovery in genome-wide experimental datasets. Nucleic Acids Res. 2019;47:D607–13. 10.1093/nar/gky1131 PMC6323986 30476243

[17] Tang Z, Li C, Kang B, Gao G, Li C, Zhang Z. GEPIA: a web server for cancer and normal gene expression profiling and interactive analyses. Nucleic Acids Res. 2017;45:W98–102. 10.1093/nar/gkx247 PMC5570223 28407145

[18] Fagerberg L, Hallström BM, Oksvold P, Kampf C, Djureinovic D, Odeberg J, et al. Analysis of the human tissue-specific expression by genome-wide integration of transcriptomics and antibody-based proteomics. Mol Cell Proteomics. 2014;13:397–406. 10.1074/mcp.M113.035600 PMC3916642 24309898

[19] Mai M, Qian C, Yokomizo A, Smith DI, Liu W. Cloning of the human homolog of conductin (AXIN2), a gene mapping to chromosome 17q23-q24. Genomics. 1999;55:341–4. 10.1006/geno.1998.5650 10049590

[20] Zu YL, Ai Y, Huang CK. Characterization of an autoinhibitory domain in human mitogen-activated protein kinase-activated protein kinase 2. J Biol Chem. 1995;270:202–6. 10.1074/jbc.270.1.202 7814374

[21] Chook YM, Blobel G. Karyopherins and nuclear import. Curr Opin Struct Biol. 2001;11:703–15. 10.1016/s0959-440x(01)00264-0 11751052

[22] Goldfarb DS, Corbett AH, Mason DA, Harreman MT, Adam SA. Importin alpha: a multipurpose nuclear-transport receptor. Trends Cell Biol. 2004;14:505–14. 10.1016/j.tcb.2004.07.016 15350979

[23] Zhou J, Dong D, Cheng R, Wang Y, Jiang S, Zhu Y, et al. Aberrant expression of KPNA2 is associated with a poor prognosis and contributes to OCT4 nuclear transportation in bladder cancer. Oncotarget. 2016;7:72767–76. 10.18632/oncotarget.11889 PMC5341943 27611951

[24] Takada T, Tsutsumi S, Takahashi R, Ohsone K, Tatsuki H, Suto T, et al. KPNA2 over-expression is a potential marker of prognosis and therapeutic sensitivity in colorectal cancer patients. J Surg Oncol. 2016;113:213–7. 10.1002/jso.24114 26663089

[25] Li XL, Jia LL, Shi MM, Li X, Li ZH, Li HF, et al. Downregulation of KPNA2 in non-small-cell lung cancer is associated with Oct4 expression. J Transl Med. 2013;11:232. 10.1186/1479-5876-11-232 PMC3849263 24070213

[26] Shi B, Su B, Fang D, Tang Y, Xiong G, Guo Z, et al. High expression of KPNA2 defines poor prognosis in patients with upper tract urothelial carcinoma treated with radical nephroureterectomy. BMC Cancer. 2015;15:380. 10.1186/s12885-015-1369-8 PMC4432830 25956057

[27] Jiang P, Tang Y, He L, Tang H, Liang M, Mai C, et al. Aberrant expression of nuclear KPNA2 is correlated with early recurrence and poor prognosis in patients with small hepatocellular carcinoma after hepatectomy. Med Oncol. 2014;31:131. 10.1007/s12032-014-0131-4 25031071

[28] Braem CV, Kas K, Meyen E, Debiec-Rychter M, Van De Ven WJ, Voz ML. Identification of a karyopherin alpha 2 recognition site in PLAG1, which functions as a nuclear localization signal. J Biol Chem. 2002;277:19673–8. 10.1074/jbc.M112112200 11882654

[29] Huang SM, Huang SP, Wang SL, Liu PY. Importin alpha1 is involved in the nuclear localization of Zac1 and the induction of p21WAF1/CIP1 by Zac1. Biochem J. 2007;402:359–66. 10.1042/BJ20061295 PMC1798434 17109628

[30] Kelly KF, Otchere AA, Graham M, Daniel JM. Nuclear import of the BTB/POZ transcriptional regulator Kaiso. J Cell Sci. 2004;117:6143–52. 10.1242/jcs.01541 15564377

[31] Sandrock K, Bielek H, Schradi K, Schmidt G, Klugbauer N. The nuclear import of the small GTPase Rac1 is mediated by the direct interaction with karyopherin alpha2. Traffic. 2010;11:198–209. 10.1111/j.1600-0854.2009.01015.x 19961560

[32] Zannini L, Lecis D, Lisanti S, Benetti R, Buscemi G, Schneider C, et al. Karyopherin-alpha2 protein interacts with Chk2 and contributes to its nuclear import. J Biol Chem. 2003;278:42346–51. 10.1074/jbc.M303304200 12909615

[33] Tseng SF, Chang CY, Wu KJ, Teng SC. Importin KPNA2 is required for proper nuclear localization and multiple functions of NBS1. J Biol Chem. 2005;280:39594–600. 10.1074/jbc.M508425200 16188882

